# Role of Melatonin in Schizophrenia

**DOI:** 10.3390/ijms14059037

**Published:** 2013-04-25

**Authors:** Armando L. Morera-Fumero, Pedro Abreu-Gonzalez

**Affiliations:** 1 Department of Internal Medicine, Dermatology and Psychiatry, School of Medicine, University of La Laguna, 38071 La Laguna, Spain; 2 Psychiatric Consultancy SC, 38001 Santa Cruz de Tenerife, Spain; 3 Department of Physiology, School of Medicine, University of La Laguna, 38071 La Laguna, Spain; E-Mail: pabreu@ull.es

**Keywords:** melatonin, schizophrenia, biological marker, therapeutic agent

## Abstract

Schizophrenia is a chronic mental disease that disturbs several cognitive functions, such as memory, thought, perception and volition. Schizophrenia’s biological etiology is multifactorial and is still under investigation. Melatonin has been involved in schizophrenia since the first decades of the twentieth century. Research into melatonin regarding schizophrenia has followed two different approaches. The first approach is related to the use of melatonin as a biological marker. The second approach deals with the clinical applications of melatonin as a drug treatment. In this paper, both aspects of melatonin application are reviewed. Its clinical use in schizophrenia is emphasized.

## 1. Introduction

Schizophrenia is a chronic and complex mental illness that disturbs several cognitive functions, such as memory, thought, perception and volition. Schizophrenia is a disease that usually evolves with outbreaks and affects approximately 0.5%–1% of the worldwide population. Its biological etiology is multifactorial and is still under investigation [1].

It is accepted that schizophrenia is not a single disease, but different subgroups of clinically and biologically heterogeneous entities [2]. It remains a matter of debate if current diagnostic armamentarium (Magnetic Resonance Imaging (MRI) and Positron Emission Tomography (PET) studies, laboratory tests, genetic studies, *etc*.) could help clinicians with the diagnosis, treatment and prognosis.

Initial ideas about the relationships between the pineal gland and psychiatric conditions stem from Descartes, who placed within the pineal gland the seat of rational thought and the link between body and soul [3]. This was the rationale to treat psychiatric diseases with extracts of pineal gland. Maybe the first paper of this type can be traced as far as the beginning of the twentieth century [4], when a group of psychiatric patients was treated with extracts of pineal glands. During the second half of the twentieth century, there was a renewed interest in the therapeutic efficacy of pineal extracts, which were administered as a treatment for psychotic states [5,6]. The main hormonal product of the pineal gland is melatonin (MLT). Approximately 80% of pineal secretion is in the form of MLT, though another product, such as 5-methoxytryptamine, is also secreted by the pineal gland [7].

The research on MLT as a biological marker of schizophrenia has gone parallel with the development of laboratory techniques that allow researchers to quantify MLT in a valid and reliable fashion. The first techniques to measure MLT were semi-quantitative, based on the capacity of MLT to bleaching tadpoles and frogs’ skin [8,9]. It is not until the late seventies of the past century that other quantitative techniques were developed for the determination of MLT in several biological fluids [10–12].

MLT research as a biological marker of schizophrenia has reported controversial results. It has been found that the blood of schizophrenic patients have increased [13,14], decreased [15,16] or unaffected [17,18] MLT levels. On the other hand, schizophrenia MLT use as a therapeutic agent has mainly been focused on sleep disorders and tardive dyskinesia treatments [19,20]. The aim of this research is to review the use of MLT in schizophrenia, in both aspects, as a biological marker and as a therapeutic agent.

In mammals, the information on environmental lighting conditions, which is neutrally perceived by the retina, is finally converted into nocturnally elevated synthesis of the principal pineal secretion product, MLT. The pineal gland is a photo-neuroendocrine organ, converting external luminous stimuli into a hormone secretion responsible for synchronizing the internal homeostasis and the environmental conditions [21]. In the photo-responsive pineal gland, the message of darkness relies on the master circadian pacemaker, the suprachiasmatic nucleus (SCN). The control of the SCN on circadian rhythmicity in peripheral tissues may be direct, neural mediated (autonomic nervous system) and indirect, hormonal-mediated (pineal MLT secretion). The circadian activity of the SCN is synchronized to the light/dark cycle mainly through light perceived by the retina [22]. In the absence of light (dark phase), the increase in MLT biosynthesis in the pineal gland is stimulated by electrical signals originating from neurons of the SCN [23]. The principal neurotransmitter at the postganglionic sympathetic nerve terminal is norepinephrine (NE) [24]. During daytime, NE release from the sympathetic fibers is suppressed by an increased electrical activity in the SCN. At night, when the SCN activity is inhibited, the release of NE is enhanced [25]. MLT is a metabolite of tryptophan (TRP). The step-limit to this metabolic route is the alkylation of serotonin by AANAT (aryl-alkylamine-N-acetyl-transferase; EC 2.3.1.87) [26].

Apart from blood, saliva and urine, MLT has been detected in the CSF of mammals and the anterior chamber of the eye [27]. MLT is also found in many fluids related to reproduction [28]. In the brain, at least in animal models, MLT has been reported to be concentrated in several regions of the cortex, cerebellum, thalamus and the paraventricular nuclei of the hypothalamus [29]. Furthermore, the highest concentration of MLT in humans is located on the upper portion of the gastrointestinal tract (GIT). MLT concentrations in GIT mucosa exceed 400-times blood levels and occur mainly after food intake, rich in proteins and high tryptophan content [30], with independence of circadian rhythms.

MLT is widely distributed along the human tissues, allowing the carrying out of its pleiotropic functions [31]. In humans, MLT roles are numerous and include, among others, control on the circadian rhythm acting as neuromodulator, hormone, cytokine and biological response mediator [32]. It also affects brain, immune, gastrointestinal, cardiovascular, renal, bone and endocrine functions and acts as a natural oncostatic and anti-aging molecule [33–36].

Regarding the possible effects of the administration of MLT in humans and the synthesis of different hormones, there are some controversies and coincidences. Most authors match a negative correlation between MLT and luteinizing hormone (LH) and testosterone [37,38]. With respect to thyroid hormones, the existing data in the literature are contradictory, an inverse correlation or a lack of correlation between MLT and thyroid hormones have been reported [37,38]. In the classic study of Seabra *et al*. [39], 40 volunteers received 10 mg of MLT during 28 days in a double-blind clinical trial. The laboratory exams included a complete blood analysis concerning hormones, such as, T4, TSH, LH, FSH and cortisol. No significant differences were observed between placebo and MLT groups.

Many MLT actions are mediated through the interaction with specific membrane bound receptors. To date, two membrane bound mammalian MLT receptors have been identified and characterized: MT_1_ or MEL_1a_ and MT_2_ or MEL_1b_ [40]. Furthermore, MLT as a lipophilic molecule can act through a non-receptor mediated mechanism. Respecting this action, the more representative property is as a radical scavenger for reactive oxygen species (ROS) and reactive nitrogen species (RNS) [41]. The oxygen or nitrogen reactive species eliminated by MLT include hydroxyl radical (OH**^•^**), hydrogen peroxide (H_2_O_2_) and free radical derivatives of nitric oxide (NO) [42,43]. In addition, MLT stimulates antioxidant enzymes activity and gene expression; three of these principal antioxidant enzymes are glutathione peroxidase (GPx), superoxide dismutase (SOD) and catalase (CAT) [44].

## 2. Role of MLT in Schizophrenia

Hallucinations and delusion are core positive symptoms of schizophrenia [45]. The first attempt to link MLT with schizophrenia was posed by McIsaac in 1961 [46] when he proposed that the chemical structure of MLT was very similar to the structure of the hallucinogenic harmala alkaloids, harmine and harmaline. The formation of such alkaloid, 10-methoxyharmalan, could be produced by the MLT removal of one molecule of water by cyclodehydration. Harmala alkaloids are potent monoaminooxidase (MAO) inhibitors and could prevent the normal breakdown of 5HT, which would cause 5HT metabolism to shut down the pathways, which would lead to the production of more 5-methoxytriptamine, MLT and 10-methoxyharmalan. By doing this, once 10-methoxyharmalan is formed, it tends to maintain (positive feedback) its own formation. To our knowledge, there is no research in humans that has backed this hypothesis.

Since the isolation of MLT in 1958 [47], there has been an increased interest in researching on MLT and psychiatry. Because the chemical isolation of MLT from pineal gland extracts took several years, early studies were carried out using extracts of pineal glands, mainly from bovine pineal glands [48]. Readily it was acknowledge that what was believed, that the pineal gland did not have a clearly defined function [49], was soon reformulated, and several researches started pointing out the MLT physiological mechanisms of action [48,50–53]. It soon became clear that there were two areas of research clearly delimited in psychiatry with respect to MLT. The first area was related to the use of MLT as a biological marker of psychiatric pathologies. The second area was related to the clinical uses of MLT as a possible psychiatric therapeutic agent. Both areas grew together and at the same time with more or less success, depending on how the knowledge on MLT functions were being achieved. With respect to schizophrenia, initial studies using injections of bovine pineal gland extracts [5,6,54] soon gave way to the use of MLT preparations to treat schizophrenia sleep disorders [19,55] and tardive dyskinesia [20,56]. In the next two sections, both aspects of MLT, as a marker and as a therapeutic agent, will be revised in depth.

## 3. MLT as a Marker of Schizophrenia

Two aspects of MLT as a marker of schizophrenia deserve to be mentioned. First, researches study MLT levels in specific time periods or just specific time points [57–59]. This kind of research is not difficult to carry out, because patients are sampled few times along the day. The second research group attempted to study parameters of the MLT circadian rhythm, such as the MESoR, the dim-light MLT onset (DLMO), the dim-light MLT offset (DLMOff) and the acrophase or the nadir, just to mention some of them. These kinds of studies need patients to be sampled several times during the day and at night [17,60]. Taking into account that these subjects are ill, sometimes acutely ill, it is not easy to obtain several measures of the biological sample (blood, urine and saliva are the most common biological fluids sampled) where MLT should be measured.

The most common reported result of MLT as a marker of the disease is that schizophrenic patients present lower night MLT levels than healthy controls [15,59–62]. A normal day/night MLT rhythm [18,63], lower early morning (07:00–08:00 am) MLT levels [58] and no difference between healthy and schizophrenic subjects [64,65] have also been reported. A possible explanation for the “low MLT syndrome” present in some schizophrenic patients may stem from the study of the enzymes involved in MLT production from 5HT, the AANAT and the hydroxyindole-*O*-methyl-transferase (HIOMT). Smith *et al*. [66] studied the pineal enzyme activity of serotonin N-acetyltransferase (SNAT) and HIOMT from the brain autopsy of 11 schizophrenic patients and 67 non-schizophrenic subjects. They found that schizophrenics compared to controls had elevated the HIOMT activity by about 25%. The authors suggest that a lack of substrate or an abnormally low activity of an enzyme prior to HIOMT in the biosynthesis of MLT could explain the observed low MLT concentrations.

MLT levels have also been used to differentiate clinical subtypes of schizophrenia. The paranoid subtype has been reported to have lower MLT levels than healthy subjects [61], as well as similar levels to healthy controls [64]. Studies carried out with other clinical subtypes include small samples [62]; therefore, any conclusion elicited from them is difficult to generalize.

The effect of antipsychotic medication on MLT has also been studied. Antipsychotic treatment has been reported to increase MLT concentrations *in vivo* [14] and *in vitro* [67]. However, the study of drug-naive (patients who never have been treated with antipsychotics) or drug-free patients is not an easy task in psychiatry because of ethical reasons. No differences in CSF of MLT concentrations among medicated and unmedicated patients and healthy controls have been reported [64]. Drug-free schizophrenic patients do not present changes in their MLT levels after being treated with typical antipsychotics [16,62]. Treatment with olanzapine, an atypical antipsychotic, did not affect the MLT circadian rhythm of a group of drug-free schizophrenic patients [18]. Quetiapine, another atypical antipsychotic, does not affect MLT secretion in healthy subjects [68]. Drug-naive, drug-withdrawn and drug-treated patients (schizophrenic and schizoaffective) on typical antipsychotics have similar levels of blood MLT concentrations [58]. We believe that the problem of studying the effect of antipsychotics on MLT levels stems from the fact that polytherapy (using several medicines at the same time) instead of monotherapy in schizophrenia treatments is the rule, not the exception [69]. In order to circumvent this problem, the conversion of the different antipsychotic doses into a chlorpromazine equivalent dose has been done by some authors [56,60,70]. This approach would help with undergoing the comparison of the different antipsychotic doses, but the information about the specificity of each antipsychotic would be lost. For example, olanzapine (an atypical antipsychotic) has been reported not to affect MLT levels in schizophrenia treated patients [18], while chlorpromazine (a typical antipsychotic) treated patients presented increased MLT concentrations [13].

Psychiatric symptoms (psychopathology) have also been related to MLT concentrations. The total score of the Brief Psychiatric Rating Scale (BPRS) correlated positively with CSF concentrations of MLT [64]. No correlations between the total score of the BPRS and MLT have also been reported [60,61]. Positive and negative symptoms, measured with the Scale for the Assessment of Positive Symptoms (SAPS) and the Scale for the Assessment of Negative Symptoms (SANS) did not correlate with the MLT Area under the Curve (AUC) [62]. No correlations between MLT levels and the SANS, SAPS and the Positive and Negative Syndrome Scale (PANSS) have also been reported [71]. Negative symptoms measured with the negative subscale of the PANSS do not correlate with MLT concentrations [18]. Summarizing, psychopathology, measured as the two big schizophrenic syndromes (positive/negative), does not seem to be related to MLT concentrations. In our opinion, the negative results may be due to the fact that the scales that psychiatrists use are more focused on the study of the schizophrenic syndromes (positive/negative) than on the study of specific symptoms. The presence/absence of correlations between MLT concentrations and total scores scales (BPRS, PANSS) is difficult to interpret, because they do not measure schizophrenic syndromes, but global symptom severity.

The number of published studies on MLT as a marker of schizophrenia circadian rhythm is scanty compared to the studies published on MLT as a non-marker of schizophrenia circadian rhythm. Furthermore, most data on MLT as a circadian rhythm marker in schizophrenia have been published on studies carried out on relatively small samples. A phase-advance of the MLT rhythm in drug-free and antipsychotic-treated schizophrenic patients has been reported [17,60,72]. Other patterns of abnormal MLT secretion have also been reported. Abnormal diurnal MLT peaks at 10:00 and 18:00 and a complete elimination of the nocturnal MLT secretion have also been reported [60]. In monozygotic twins discordant for schizophrenia, the twin with schizophrenia presented a lower MLT production and a low threshold of DLMO (DLMO < 3 pg/mL) compared to the non-affected twin, who presented a normal nocturnal rise [73]. However in another study published one year later [65] the same authors reported no differences between schizophrenic patients and healthy subjects in the DLMO, as well as the MLT level measured hourly in saliva from 20:00 to 23:00 pm. Finally, a recent paper [70] reported the existence of two subgroups of paranoid schizophrenia patients. Subgroup I had a similar pattern of MLT secretion to the control group, while subgroup II had a phase advance of MLT secretion compared to the MLT phase of the healthy subjects. Table 1 summarizes the role of MLT as a schizophrenia marker.

Current clinical information of MLT as a schizophrenia biological marker is controversial. In our opinion, future research on MLT as a marker of schizophrenia should take into account the following recommendations:

1. Mixing clinical diagnosis (schizophrenia, schizoaffective and schizophrenia spectrum disorders) should be avoided. From the clinical point of view, although the aforementioned diagnoses are psychiatric, schizoaffective disorders are subdivided into other types (manic, depressive and mixed), while schizophrenia spectrum disorders comprise different categories (schizophreniform disorder, delusional disorder, schizotypal, schizoid and paranoid ersonality disorders). Therefore, having the most homogeneous clinical diagnosis would help to clarify the relationships between schizophrenia and MLT.
2. In order to know the possible differences in MLT among schizophrenia clinical types, studies should comprise all schizophrenia subtypes. Because paranoid schizophrenia is the most prevalent subtype of schizophrenia, efforts should be made in order to gather the less prevalent schizophrenia subtypes (e.g., catatonic, hebephrenic, residual, *etc*.). No conclusion can be reached in this point if the studies comprising those subtypes of schizophrenia contain only a few subjects.
3. Research studies should not only focus on psychotic syndromes, but on psychotic symptoms. Reporting total scores give us a global idea about the intensity of the psychopathology, but the study of specific symptoms (delusions, hallucinations, blunt affect, *etc*.) would add more valuable information than just a global measure that has a more difficult interpretation from the clinical point of view. Additionally, the problem of using different scales to measure psychopathology would be solved, at least in part, because individual symptoms could be compared without the interference of the use of different scales.
4. Clinical studies should focus on total MLT measures (urine is the less invasive) rather than circadian rhythms, since they are more complex in their implementation. As far as we are concerned, the clinical setting, as inpatients or outpatients, is a difficult environment where continuous interventions (collecting regularly biological samples) are a difficult task to perform, because of the mental state of the patients. In our experience, unless there is a specific interest on studying circadian parameters (DLMO, DLMOff, acrophase, *etc*.), a global measure of MLT, for example, urine collection every 8 or 12 h, is a reasonable measure of MLT production that can be easily used in the clinical context (as a biological marker of symptoms or as a marker of treatment response).
5. When possible, the use of monotherapy is preferable to the use polytherapy. Furthermore, the control of other psychiatric medication, apart from antipsychotics, should be considered, because antidepressants, benzodiazepines and mood stabilizers may affect MLT concentrations.
6. More general methodological issues (the control of body posture, light exposure, anticoagulants used in collecting blood samples, *etc*.) should be included in the protocol study.
7. Finally, non-compliance is a big problem in psychiatry (treatments, diets, appointments, *etc*.). Non-compliance with the sampling protocol should be seriously taken into account when carrying out this kind of studies. In an outpatient context, special controls with the sampling procedure should be considered.

## 4. MLT as a Therapeutic Agent in Schizophrenia

The use of MLT as a therapeutic agent can be traced as far as 1920, when a group of patients with “dementia praecox” was treated with pineal extracts [4]. Between the decades of 1950 until the middle of the seventies, MLT was used in the form of pineal extract injections [5,54]. Several review papers have been published on MLT and psychiatry [3,74], but very few about the use of MLT as a therapeutic agent in psychiatry [75,76].

One of earliest mechanisms of MLT action was related to its hypnogenic properties [77]. Apart from the hypnogenic properties, MLT also has resynchronizing actions. MLT has been used to treat free-running rhythm disorders [78], jet lag [79] and delayed sleep phase syndrome [80], among other circadian rhythm disorders.

Several of the studies on the therapeutic effect of MLT in schizophrenia have been carried out on the MLT use for the treatment of sleep disorders. The effect of 2 mg of control released MLT on several sleep parameters in schizophrenic patients who complained of poor sleep quality and met DSM-IV insomnia criteria was studied [19]. Sleep efficiency (percentage of total time asleep over total time in bed) improved significantly after MLT treatment compared to placebo treatment. However, sleep latency (amount of time it takes to fall asleep after the lights have been turned off), total sleep time (time spent asleep after sleep onset), wake after sleep onset duration (mid-sleep arousal time after sleep onset), fragmentation index (percentage of quiet episodes that are shorter than 1 m over the total number of quiet episodes during time in bed) and the number of awakenings (the total number of awakenings during sleep) did not differ significantly from placebo. The effect of 3 mg of MLT in patients with paranoid schizophrenia who complained of initial insomnia was also studied [55]. MLT-treated patients showed a significant reduction in the number of nighttime awakenings and slept longer than the placebo treated patients did. Additionally, according to the sleep questionnaires results, subjects taking MLT compared to placebo significantly reduced sleep latency, improved quality and depth of sleep and experienced greater early-morning freshness. MLT was also used in the study of the First Night Effect (FNE) (tendency for individuals to sleep worse than normal during their first night of polysomnographic sleep evaluation) in chronic schizophrenia patients [81]. Placebo or 2 mg of control released MLT was given to patients before a polysomnographic study in two consecutive days. Compared to placebo, MLT treatment increased rapid eye movement (REM) sleep latency, decreased sleep efficiency and the duration of wakefulness during sleep was lower on the first night than on the second night. These effects were not found when the patients received a placebo. This paper results show that MLT treatment exaggerates FNE in patients with chronic schizophrenia.

MLT has direct and indirect antioxidant properties [82–84]. Because schizophrenic patients are biochemically more oxidized [85–87], the low MLT levels might be the result of the body reaction trying to compensate the hyperoxidative status. Earlier works of Altschule *et al*. [48,88] reported that schizophrenic patients had low levels of glutathione, and the injections of pineal extracts corrected those deficits [48]. Based on the results of *in vitro* studies, in which MLT reduced by about 83% the enzymatic oxidation of dopamine (DA) and by about 35.7% the autoxidation of DA, Hartley and Smith [89] proposed that MLT may act as a free radical scavenger, thus slowing down the autoxidation rate.

Tardive dyskinesia (TD) is a late-onset side effect associated with typical antipsychotic treatment. Within the first five years of exposure to typical antipsychotics, it is estimated that 3%–5% of patients will develop TD. The prevalence of TD has been estimated between 15% and 20% [90]. The pathophysiology of TD is not well defined. It has been suggested that TD is due to an increase in oxidative damage caused by free radical generation [91]. Three clinical studies have been carried out to evaluate the efficacy of MLT as antioxidant in the treatment of TD [20,56,92]. Several doses of MLT, as well as two different formulations have been studied. Two studies used control released MLT, 2 or 10 mg [20,92], while one study used 20 mg of fast released MLT [56]. Low doses of MLT do not produce an improvement of abnormal movements, while higher doses produce an amelioration of abnormal movements. Animal studies point to the same direction. Rats chronically treated with haloperidol developed abnormal oral movements referred to as vacuous chewing movements (an animal model of TD). The number of vacuous chewing movements was reversed in a MLT dose-dependent fashion (1, 2 and 5 mg/kg) [93].

Benzodiazepines (BZD) augmentation is not unusual in schizophrenia treatment in spite of the lack of evidence of its utility [94]. The use of BZD is not free of risk, dependence being one of them [95]. MLT has been used for the detoxification of BZD dependence [96,97]. There is an approved clinical trial designed to study BZD discontinuation with MLT in patients with schizophrenia [98]. As far as we know, the only paper published in which a MLT agonist (agomelatine) had been used to suspend BZD treatment in a schizophrenic patient treated with antipsychotics and a high dose of diazepam was published in 2010 [99]. Therefore, the small number of studies published on BZD discontinuation in schizophrenia precludes any definitive conclusion. We are looking forward to the results of the clinical trial aforementioned. Table 2 summarizes the role of MLT as a schizophrenia therapeutic agent.

From our point of view, future research on MLT as a treatment option in schizophrenia should attempt to answer the following questions:

1. Which dose/s is/are therapeutic?
2. Are there specific aspects of the disease (symptoms, clinical subtypes, complications, *etc*.) that could benefit from the MLT treatment?
3. The different formulations of MLT, e.g., slow/control release, fast release or a combination of both, are equally effective?

The small quantity of clinical studies that have been published about the use of MLT as a therapeutic agent in schizophrenia is surprising. In a recent review about human trials in which the clinical use of MLT was evaluated [100], there is no reference to the role of MLT as a therapeutic agent in schizophrenia.

## 5. Conclusions and Future Directions

In summary, despite that MLT was discovered and isolated more than fifty years ago [47], the results from studies relating MLT to schizophrenia appear to be rather inconclusive. It is noteworthy that the use of MLT as a medicine was approved by the European Medicines Agency in 2007 [101], but the research on its therapeutic applications is very scanty compared to the research carried out on the role of MLT as marker of schizophrenia. At present, MLT can be measured with easy and inexpensive biochemical techniques. As far as we know, no important deleterious effects have been reported in humans when MLT has been administered in the clinical context [39,102]. It is probable that when MLT use would be more generalized in clinical practice, MLT measurement would become a routine test and costs would be even cheaper. With the recent advent of new drugs targeting MLT receptors (ramelteon, agomelatine, tasimelteon, *etc*.). We will likely assist in the coming years an explosion affecting this area of research, in basic research, clinical trials and new clinical indications.

## Figures and Tables

**Table 1:** Role of melatonin as a marker of schizophrenia.

Use	References
Biological marker of the disease	[15,18,57–65]
Clinical schizophrenia subtypes	[61,62,64]
Antipsychotic effect	[13,14,16,18,56,58,60,62,64,70]
Psychiatric symptoms	[18,60–62,64,71]
Circadian rhythms	[17,60,65,70,72,73]

**Table 2:** Role of melatonin as a schizophrenia therapeutic agent.

Treatment	References
Schizophrenia sleep disorders	[19,55,81]
Tardive dyskinesia	[20,56,92]
Benzodiazepines discontinuation	[99]

## References

[1] Freedman R. (2003). Schizophrenia. N. Engl. J. Med.

[2] Wyatt R.J., Potkin S.G., Kleinman J.E., Weinberger D.R., Luchins D.J., Jeste D.V. (1981). The schizophrenia syndrome. Examples of biological tools for subclassification. J. Nerv. Ment. Dis.

[3] Mullen P.E., Silman R.E. (1977). The pineal and psychiatry: A review. Psychol. Med.

[4] Becker W.H. (1920). Epiglandol bei dementia praecox. Ther. Halbmonast.

[5] Altschule M.D. (1957). Some effects of aqueous extracts of acetone-dried beef-pineal substance in chronic schizophrenia. N. Engl. J. Med.

[6] Eldred S.H., Bell N.W., Sherman L.J. (1960). A pilot study comparing the effects of pineal extract and a placebo in patients with chronic schizophrenia. N. Engl. J. Med.

[7] Hardeland R. (2010). Melatonin metabolism in the central nervous system. Curr. Neuropharmacol.

[8] McCord C.P., Allen F.P. (1917). Evidences associating pineal gland function with alterations in pigmentation. J. Exp. Zool.

[9] Bors O., Ralston W.C. (1951). A simple assay of mammalian pineal extracts. Proc. Soc. Exp. Biol. Med.

[10] Lewy A.J., Markey S.P. (1978). Analysis of melatonin in human plasma by gas chromatography negative chemical ionization mass spectrometry. Science.

[11] Lynch H.J., Ozaki Y., Wurtman R.J. (1978). The measurement of melatonin in mammalian tissues and body fluids. J. Neural Transm. Suppl.

[12] Thoresen T.S. (1978). Radioimmunoassay for melatonin in human serum. Scand. J. Clin. Lab. Investig.

[13] Smith J.A., Mee T.J., Barnes J.L. (1977). Elevated melatonin serum concentrations in psychiatric patients treated with chlorpromazine. J. Pharm. Pharmacol.

[14] Smith J.A., Barnes J.L., Mee T.J. (1979). The effect of neuroleptic drugs on serum and cerebrospinal fluid melatonin concentrations in psychiatric subjects. J. Pharm. Pharmacol.

[15] Fanget F., Claustrat B., Dalery J., Brun J., Terra J.L., Marie-Cardine M., Guyotat J. (1989). Nocturnal plasma melatonin levels in schizophrenic patients. Biol. Psychiatry.

[16] Robinson S., Rosca P., Durst R., Shai U., Ghinea C., Schmidt U., Nir I. (1991). Serum melatonin levels in schizophrenic and schizoaffective hospitalized patients. Acta Psychiatr. Scand.

[17] Rao M.L., Gross G., Strebel B., Halaris A., Huber G., Braunig P., Marler M. (1994). Circadian rhythm of tryptophan, serotonin, melatonin, and pituitary hormones in schizophrenia. Biol. Psychiatry.

[18] Mann K., Rossbach W., Muller M.J., Muller-Siecheneder F., Pott T., Linde I., Dittmann R.W., Hiemke C. (2006). Nocturnal hormone profiles in patients with schizophrenia treated with olanzapine. Psychoneuroendocrinology.

[19] Shamir E., Laudon M., Barak Y., Anis Y., Rotenberg V., Elizur A., Zisapel N. (2000). Melatonin improves sleep quality of patients with chronic schizophrenia. J. Clin. Psychiatry.

[20] Shamir E., Barak Y., Shalman I., Laudon M., Zisapel N., Tarrasch R., Elizur A., Weizman R. (2001). Melatonin treatment for tardive dyskinesia: A double-blind, placebo-controlled, crossover study. Arch. Gen. Psychiatry.

[21] Csernus V., Mess B. (2003). Biorhythms and pineal gland. Neuro Endocrinol. Lett.

[22] Morin L.P., Allen C.N. (2006). The circadian visual system. Brain Res. Rev.

[23] Moore R.Y. (1996). Neural control of the pineal gland. Behav. Brain Res.

[24] Klein D.C., Weller J.L., Moore R.Y. (1971). Melatonin metabolism: Neural regulation of pineal serotonin: Acetyl coenzyme a N-acetyl-transferase activity. Proc. Nat. Acad. Sci. USA.

[25] Klein D.C., Schaad N.L., Namboordiri M.A., Yu L., Weller J.L. (1992). Regulation of pineal serotonin N-acetyltransferase activity. Biochem. Soc. Trans.

[26] Klein D.C., Coon S.L., Roseboom P.H., Weller J.L., Bernard M., Gastel J.A., Zatz M., Iuvone P.M., Rodriguez I.R., Bégay V. (1997). The melatonin rhythm-generating enzyme: Molecular regulation of serotonin N-acetyltransferase in the pineal gland. Recent Prog. Horm. Res.

[27] Martin X.D., Malina H.Z., Brennan M.C., Hendrikson P.H., Lichter P.R. (1992). The ciliary body the third organ found to synthesize indoleamines in humans. Eur. J. Ophthalmol.

[28] Cagnacci A. (1996). Melatonin in relation to physiology in adult humans. J. Pineal Res.

[29] Menendez-Pelaez A., Reiter R.J. (1993). Distribution of melatonin in mammalian tissues: The relative importance of nuclear versus cytosolic localization. J. Pineal Res.

[30] Konturek S.J., Konturek P.C., Brzozowski T., Bubenik G.A. (2007). Role of melatonin in upper gastrointestinal tract. J. Physiol. Pharmacol.

[31] Radogna F., Diederich M., Ghibelli L. (2010). Melatonin: A pleiotropic molecule regulating inflammation. Biochem. Pharmacol.

[32] Reiter R.J. (1991). Melatonin: The chemical expression of darkness. Mol. Cell. Endocrinol.

[33] Leja-Szpak A., Jaworek J., Pierzchalski P., Reiter R.J. (2010). Melatonin induces pro-apoptotic signaling pathway in human pancreatic carcinoma cells (PANC-1). J. Pineal Res.

[34] Dominguez-Rodriguez A., Abreu-Gonzalez P., Sanchez-Sanchez J.J., Kaski J.C., Reiter R.J. (2010). Melatonin and circadian biology in human cardiovascular disease. J. Pineal Res.

[35] Hardeland R. (2012). Neurobiology, pathophysiology, and treatment of melatonin deficiency and dysfunction. Sci. World J..

[36] Sanchez-Barcelo E.J., Mediavilla M.D., Alonso-Gonzalez C., Reiter R.J. (2012). Melatonin uses in oncology: Breast cancer prevention and reduction of the side effects of chemotherapy and radiation. Expert Opin. Investig. Drugs.

[37] Bellipanni G., Bianchi P., Pierpaoli W., Bulian D., Llya E. (2001). Effects of melatonin in perimenopausal and menopausal women: A randomized and placebo controlled study. Exp. Gerontol.

[38] Nonomiya T., Iwatani N., Tomoda A., Miike T. (2001). Effects of exogenous melatonin on pituitary hormones in humans. Clin. Physiol.

[39] Seabra M.L., Bignotto M., Pinto L.R., Tufik S. (2000). Randomized, double-blind clinical trial, controlled with placebo, of the toxicology of chronic melatonin treatment. J. Pineal Res.

[40] Dubocovich M.L., Delagrange P., Krause D.N., Sugden D., Cardinali D.P., Olcese J. (2010). International Union of Basic and Clinical Pharmacology. LXXV. Nomenclature, classification, and pharmacology of G protein-coupled melatonin receptors. Pharmacol. Rev.

[41] Tan D.X., Manchester L.C., Terron M.P., Flores L.J., Reiter R.J. (2007). One molecule, many derivatives: A never-ending interaction of melatonin with reactive oxygen and nitrogen species?. J. Pineal Res.

[42] Galano A., Tan D.X., Reiter R.J. (2011). Melatonin as a natural ally against oxidative stress: A physicochemical examination. J. Pineal Res.

[43] Tan D.X., Manchester L.C., Hardeland R., Lopez-Burillo S., Mayo J.C., Sainz R.M., Reiter R.J. (2003). Melatonin: A hormone, a tissue factor, an autocoid, a paracoid, and an antioxidant vitamin. J. Pineal Res.

[44] Rodriguez C., Mayo J.C., Sainz R.M., Antolín I., Herrera F., Martín V., Reiter R.J. (2004). Regulation of antioxidant enzymes: A significant role for melatonin. J. Pineal Res.

[45] Rosen C., Grossman L.S., Harrow M., Bonner-Jackson A., Faull R. (2011). Diagnostic and prognostic significance of schneiderian first-rank symptoms: A 20-year longitudinal study of schizophrenia and bipolar disorder. Compr. Psychiatry.

[46] McIsaac W.M. (1961). A biochemical concept of mental disease. Postgrad. Med.

[47] Lerner A.B., Case J.D., Takahashi Y., Lee T.H., Mori W. (1958). Isolation of melatonin, the pineal gland factor that lightens melanocytes. J. Am. Chem. Soc.

[48] Altschule M.D., Siegel E.P., Goncz R.M., Murname J.P. (1954). Effect of pineal extracts on blood glutathione level in psychotic patients. AMA Arch. Neurol. Psychiatry.

[49] Miles A., Philbrick D.R. (1988). Melatonin and psychiatry. Biol. Psychiatry.

[50] Barchas J. (1968). Studies on the relation of melatonin to sleep. Proc. West. Pharmacol. Soc.

[51] Hipkin L.J. (1970). Effect of 5-methoxytryptophol and melatonin on uterine weight responses to human chorionic gonadotrophin. J. Endocrinol.

[52] Smythe G.A., Lazarus L. (1974). Suppression of human growth hormone secretion by melatonin and cyproheptadine. J. Clin. Investig.

[53] Nordlund J.J., Lerner A.B. (1977). The effects of oral melatonin on skin color and on the release of pituitary hormones. J. Clin. Endocrinol. Metab.

[54] Bigelow L.B. (1974). Effects of aqueous pineal extract in chronic schizophrenia. Biol. Psychiatry.

[55] Suresh Kumar P.N., Andrade C., Bhakta S.G., Singh N.M. (2007). Melatonin in schizophrenic outpatients with insomnia: A double-blind, placebo-controlled study. J. Clin. Psychiatry.

[56] Castro F., Carrizo E., Prieto de R.D., Rincon C.A., Asian T., Medina-Leendertz S., Bonilla E. (2011). Effectiveness of melatonin in tardive dyskinesia. Invest. Clin.

[57] Diaz-Mesa E., Morera-Fumero A.L., Abreu-Gonzalez P., Jimenez-Sosa A., Henry M., Fernandez-Lopez L., Intxausti A., Gracia-Marco R. (2010). Seasonality of serum melatonin concentrations in paranoid schizophrenic inpatients. Eur. Neuropsychopharmacol.

[58] Rao M.L., Gross G., Strebel B., Braunig P., Huber G., Klosterkotter J. (1990). Serum amino acids, central monoamines, and hormones in drug-naive, drug-free, and neuroleptic-treated schizophrenic patients and healthy subjects. Psychiatry Res.

[59] Ferrier I.N., Johnstone E.C., Crow T.J., Arendt J. (1982). Melatonin/cortisol ratio in psychiatric illness. Lancet.

[60] Jiang H.K., Wang J.Y. (1998). Diurnal melatonin and cortisol secretion profiles in medicated schizophrenic patients. J. Formos. Med. Assoc.

[61] Monteleone P., Maj M., Fusco M., Kemali D., Reiter R.J. (1992). Depressed nocturnal plasma melatonin levels in drug-free paranoid schizophrenics. Schizophr. Res.

[62] Monteleone P., Natale M., La Rocca A., Maj M. (1997). Decreased nocturnal secretion of melatonin in drug-free schizophrenics: No change after subchronic treatment with antipsychotics. Neuropsychobiology.

[63] Vigano D., Lissoni P., Rovelli F., Roselli M.G., Malugani F., Gavazzeni C., Conti A., Maestroni G. (2001). A study of light/dark rhythm of melatonin in relation to cortisol and prolactin secretion in schizophrenia. Neuro Endocrinol. Lett.

[64] Beckmann H., Wetterberg L., Gattaz W.F. (1984). Melatonin immunoreactivity in cerebrospinal fluid of schizophrenic patients and healthy controls. Psychiatry Res.

[65] Afonso P., Figueira M.L., Paiva T. (2011). Sleep-promoting action of the endogenous melatonin in schizophrenia compared to healthy controls. Int. J. Psychiatry Clin. Pract.

[66] Smith J.A., Mee T.J., Padwick D.J., Spokes E.G. (1981). Human post-mortem pineal enzyme activity. Clin. Endocrinol.

[67] Nir I., Hirschmann N. (1983). Effect of psychotrophic drugs on [14C]tryptophan metabolism in cultured rat pineal gland. Biochem. Pharmacol.

[68] Cohrs S., Pohlmann K., Guan Z., Jordan W., Meier A., Huether G., Ruther E., Rodenbeck A. (2004). Quetiapine reduces nocturnal urinary cortisol excretion in healthy subjects. Psychopharmacology.

[69] Tani H., Uchida H., Suzuki T., Fujii Y., Mimura M. (2013). Interventions to reduce antipsychotic polypharmacy: A systematic review. Schizophr. Res.

[70] Wulff K., Dijk D.J., Middleton B., Foster R.G., Joyce E.M. (2012). Sleep and circadian rhythm disruption in schizophrenia. Br. J. Psychiatry.

[71] Bersani G., Mameli M., Garavini A., Pancheri P., Nordio M. (2003). Reduction of night/day difference in melatonin blood levels as a possible disease-related index in schizophrenia. Neuro Endocrinol. Lett.

[72] Wirz-Justice A., Cajochen C., Nussbaum P. (1977). A schizophrenic patient with an arrhythmic circadian rest-activity cycle. Psychiatry Res.

[73] Afonso P., Brissos S., Figueira M.L., Paiva T. (2010). Discrepant nocturnal melatonin levels in monozygotic twins discordant for schizophrenia and its impact on sleep. Schizophr. Res.

[74] Pacchierotti C., Iapichino S., Bossini L., Pieraccini F., Castrogiovanni P. (2001). Melatonin in psychiatric disorders: A review on the melatonin involvement in psychiatry. Front. Neuroendocrinol.

[75] Morera A., Henry M., Abreu P., Gracia R. (2006). Melatonin therapeutic use in psychiatry: A 39 year bibliographic study. Actas Esp Psiquiatr.

[76] Maldonado M.D., Reiter R.J., Perez-San-Gregorio M.A. (2009). Melatonin as a potential therapeutic agent in psychiatric illness. Hum. Psychopharmacol.

[77] Lushington K., Pollard K., Lack L., Kennaway D.J., Dawson D. (1997). Daytime melatonin administration in elderly good and poor sleepers: Effects on core body temperature and sleep latency. Sleep.

[78] Arendt J., Aldhous M., Wright J. (1988). Synchronisation of a disturbed sleep-wake cycle in a blind man by melatonin treatment. Lancet.

[79] Arendt J., Aldhous M., Marks V. (1986). Alleviation of jet lag by melatonin: Preliminary results of controlled double blind trial. BMJ.

[80] Kayumov L., Brown G., Jindal R., Buttoo K., Shapiro C.M. (2001). A randomized, double-blind, placebo-controlled crossover study of the effect of exogenous melatonin on delayed sleep phase syndrome. Psychosom. Med.

[81] Shamir E., Rotenberg V.S., Laudon M., Zisapel N., Elizur A. (2000). First-night effect of melatonin treatment in patients with chronic schizophrenia. J. Clin. Psychopharmacol.

[82] Antolin I., Rodriguez C., Sainz R.M., Mayo J.C., Uria H., Kotler M.L., Rodriguez-Colunga M.J., Tolivia D., Menendez-Pelaez A. (1996). Neurohormone melatonin prevents cell damage: Effect on gene expression for antioxidant enzymes. FASEB J.

[83] Tan D.X., Manchester L.C., Reiter R.J., Plummer B.F. (1999). Cyclic 3-hydroxymelatonin: A melatonin metabolite generated as a result of hydroxyl radical scavenging. Biol. Signals Recept.

[84] Millan-Plano S., Piedrafita E., Miana-Mena F.J., Fuentes-Broto L., Martinez-Ballarin E., Lopez-Pingarron L., Saenz M.A., Garcia J.J. (2010). Melatonin and structurally-related compounds protect synaptosomal membranes from free radical damage. Int. J. Mol. Sci.

[85] Dietrich-Muszalska A., Olas B., Rabe-Jablonska J. (2005). Oxidative stress in blood platelets from schizophrenic patients. Platelets.

[86] Yao J.K., Leonard S., Reddy R. (2006). Altered glutathione redox state in schizophrenia. Dis. Markers.

[87] Korotkova E.I., Misini B., Dorozhko E.V., Bukkel M.V., Plotnikov E.V., Linert W. (2011). Study of OH radicals in human serum blood of healthy individuals and those with pathological schizophrenia. Int. J. Mol. Sci.

[88] Altschule M.D., Siegel E.P., Hennneman D.H. (1952). Blood glutathione level in mental disease before and after treatment. AMA Arch. Neurol. Psychiatry.

[89] Hartley R., Smith J.A. (1972). Inhibition of catecholamine oxidation by indoles. Biochem. Pharmacol.

[90] Kane J.M., Woerner M., Lieberman J. (1985). Tardive dyskinesia: Prevalence, incidence, and risk factors. Psychopharmacology.

[91] Cadet J.L., Kahler L.A. (1994). Free radical mechanisms in schizophrenia and tardive dyskinesia. Neurosci. Biobehav. Rev.

[92] Shamir E., Barak Y., Plopsky I., Zisapel N., Elizur A., Weizman A. (2000). Is melatonin treatment effective for tardive dyskinesia?. J. Clin. Psychiatry.

[93] Naidu P.S., Singh A., Kaur P., Sandhir R., Kulkarni S.K. (2003). Possible mechanism of action in melatonin attenuation of haloperidol-induced orofacial dyskinesia. Pharmacol. Biochem. Behav.

[94] Volz A., Khorsand V., Gillies D., Leucht S. (2007). Benzodiazepines for schizophrenia. Cochrane Database Syst. Rev..

[95] Petursson H., Lader M.H. (1981). Withdrawal from long-term benzodiazepine treatment. Br. Med. J.

[96] Dagan Y., Zisapel N., Nof D., Laudon M., Atsmon J. (1997). Rapid reversal of tolerance to benzodiazepine hypnotics by treatment with oral melatonin: A case report. Eur. Neuropsychopharmacol.

[97] Garfinkel D., Zisapel N., Wainstein J., Laudon M. (1999). Facilitation of benzodiazepine discontinuation by melatonin: A new clinical approach. Arch. Intern. Med.

[98] Baandrup L., Fagerlund B., Jennum P., Lublin H., Hansen J.L., Winkel P., Gluud C., Oranje B., Glenthoj B.Y. (2011). Prolonged-release melatonin versus placebo for benzodiazepine discontinuation in patients with schizophrenia: A randomized clinical trial—The SMART trial protocol. BMC Psychiatry.

[99] Morera-Fumero A.L., Diaz-Mesa E., Abreu-Gonzalez P., Henry M., Yelmo S., Fernandez-Lopez L., Gracia-Marco R. (2010). Agomelatine facilitates benzodiazepine discontinuation in schizophrenia with severe insomnia. Eur. Psychiatry.

[100] Sanchez-Barcelo E.J., Mediavilla M.D., Tan D.X., Reiter R.J. (2010). Clinical uses of melatonin: Evaluation of human trials. Curr. Med. Chem.

[101] European Medicines Agency (EMA) (2007). Melatonin.

[102] Morera A.L., Henry M., de La Varga M. (2001). Safety in melatonin use. Actas Esp Psiquiatr.

